# A method for phenotyping lettuce volume and structure from 3D images

**DOI:** 10.1186/s13007-025-01347-y

**Published:** 2025-02-24

**Authors:** Victor Bloch, Alexey Shapiguzov, Titta Kotilainen, Matti Pastell

**Affiliations:** 1 Production Systems Unit, Natural Resources, Institute Luke (Finland), 00790 Helsinki, Finland; 2 Agricultural and Biosystems Engineering, Agricultural Research Organization (ARO), the Volcani Centre, P.O. Box 15159, 7505101 Rishon LeZion, Israel

**Keywords:** Lettuce fresh weight, Lettuce 3D modelling, Plant rosette structure

## Abstract

Monitoring plant growth is crucial for effective crop management, and using color and depth (RGBD) cameras to model lettuce has emerged as one of the most convenient and non-invasive methods. In recent years, deep learning techniques, particularly neural networks, have become popular for estimating lettuce fresh weight. However, these models are typically specific to particular datasets, lack domain adaptation, and are often limited by the availability of open-access datasets. In this study, we propose a method based on plant geometric features for estimating the rosette structure and volume of lettuce. This new approach was compared to existing methods that reconstruct surfaces from point clouds, such as Ball Pivoting and Alpha Shapes. The proposed method creates a tight hull around the plant's point cloud, preserving high detail of the rosette structure while filling in surface holes in areas not visible to 3D cameras. Using a linear regression model, we estimated fresh weight for this dataset, achieving a root mean square error (RMSE) of 18.2 g when using only the estimated plant volume, and 17.3 g when both volume and geometric features were included. Additionally, we introduced new geometric features that characterize leaf density, which could be useful for breeding applications. A dataset of 402 point clouds of lettuce plants, captured before harvest, was compiled using one top-down and three side-view 3D cameras.

## Background

Monitoring plant health and modeling their structure during growth are essential for plant research, breeding, and optimizing production [1, 2]. Computer vision and deep learning algorithms are increasingly used to optimize plant spacing during growth, determine the ideal harvest time [3], and efficiently manage resources like water and electricity to meet crop demands [4, 5].

Several non-destructive methods for plant monitoring and modeling are currently in use. Photogrammetry, for example, reconstructs 3D point clouds from processed RGB images. While regular RGB cameras can be used, this method typically requires a large number of images and significant processing time. For instance, Kochi et al. [6] used up to 72 images and 3 h of processing time to create detailed plant models in a greenhouse, while Salter et al. [7] used 120 images with a processing time of 5–10 min. Andújar et al. [8] modeled weeds in field conditions using 40–50 images per plant.

In contrast, 3D cameras offer the advantage of fast data capture, providing both RGB and depth (RGBD) frames and point clouds of plants in real time. For commercial applications, 3D cameras are typically installed above plants, providing only a top-down view. Zhang et al. [9] employed a point cloud processing technique and developed the PointCNN method for predicting fresh weight (FW), while Lou et al. [10] used geometric methods to complete incomplete lettuce point clouds for FW estimation. Mortensen et al. [11] also used image and point cloud processing to estimate FW in field-grown plants.

To calculate geometric features, such as volume, from plant 3D models, the plant’s surface is typically reconstructed. Various methods for surface reconstruction from 3D point clouds have been reviewed by Fei et al. [12] and Sulzer et al. [13]. Sulzer et al. [13] tested widely used methods (such as Ball Pivoting and Poisson Surface Reconstruction) alongside newly developed neural network (NN) techniques, using a benchmark of datasets e.g., Wu et al. [14]. Notably, none of these studies addressed fractal-shaped objects, such as lettuce plants, either in their training or validation datasets.

Common lettuce traits estimated for monitoring and breeding purposes include fresh weight, dry weight, plant height, diameter, and leaf area [9]. However, these traits do not provide information about the rosette structure, such as leaf density and distribution. These geometric features are not only valuable for breeding but also crucial for more accurate FW estimation, as leaf distribution can vary significantly between plants.

A more “black-box” approach to predicting plant FW and other parameters relies on RGBD images and uses convolutional neural networks (CNNs). To advance FW prediction methods, the Autonomous Greenhouse Challenge [15] created a dataset of plant RGBD images that has been used in several studies. Zhang et al. [16] applied a custom CNN to several plant types, while Gang et al. [17] used a two-stage CNN architecture based on ResNet50V2 for RGBD images. Zhang et al. [18] implemented a three-stage CNN, and Lin et al. [19] combined CNNs with plant geometric features. Buxbaum et al. [20] employed a ResNet50-based NN in commercial greenhouse conditions. Additionally, FW was estimated without depth data by Reyes-Yanes et al. [21], who used two RGB images and Mask-RCNN, and by Tan et al. [22], who developed PosNet for early growth stages.

Reported results suggest that CNN-based algorithms using RGBD images provide the most accurate FW predictions. However, their performance is highly dependent on the training data, which is often limited to only a few hundred samples—too few to build models that are generalizable across different datasets [23]. To our knowledge, only a few studies [9] have conducted applicability analyses, using images from independent trials to validate these models externally, or attempted domain adaptation, as seen in other agricultural tasks Magistri et al. [24]. Moreover, the only found freely available dataset is the one provided by the Autonomous Greenhouse Challenge [15].

The challenges of plant modeling in computer vision are exacerbated by environmental variables and plant characteristics [3]. Due to the lack of available data and the difficulty in adapting pre-existing neural networks to new domains, this study focuses on volume estimation based on plant 3D model reconstruction using physically explainable features. We propose a novel method for detailed surface reconstruction of the lettuce rosette using an interpolation technique. Our study focuses on lettuce (Lactuca sativa L.), a commercially important leafy vegetable grown in controlled environments such as greenhouses and vertical farms [25]. We designed a simple measuring setup constructed from off the shelf components, and created a dataset point clouds of plant at the harvesting stage.

## Methods

### Plant material and growing conditions

Three consecutive experiments were carried out in a greenhouse and vertical farming research unit at the Natural Resources Institute Finland (Luke), Horticulture Research Station in Piikkiö, Finland. Lettuce variety ‘Katusa’ was sown in pots, with one seed per pot, using Kekkilä VHM 620 AirBoost lettuce peat (Kekkilä-BVB, Vantaa, Finland). A total of 132 pots were used in each experimental round. After a two-week seedling period, the lettuce plants were transferred to a controlled-environment experimental unit. The plants were grown using a nutrient film technique (NFT) system under LED lights (Valoya BX120 Solray, Valoya Oy, Helsinki, Finland).

### Measuring system

**Fig. 1:**
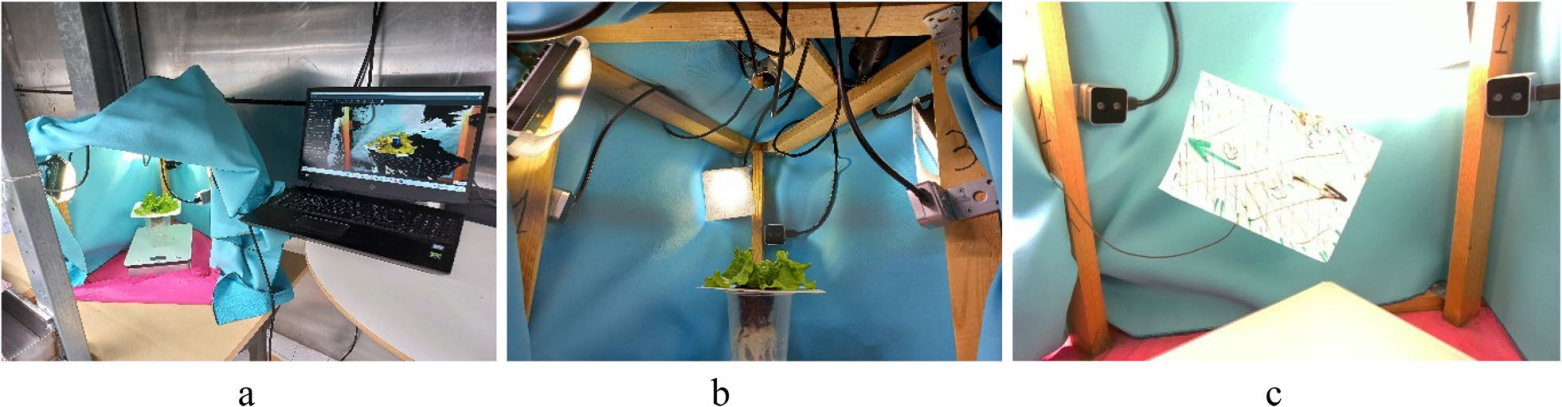
Structure of the plant 3D recording system. A frame with four 3D cameras installed on the sides and on the top of a lettuce plant and the lights: general view of the recording setup (**a**), side view from the camera 1 (**b**), calibration sheet (**c**)

Three-dimensional imaging of lettuce was performed using a frame that held four 3D cameras (D405, Intel RealSense, California, USA) with the depth accuracy of ± 2% at 50 cm, positioned on three sides and above the plant, as shown in Fig. 1. The depth field of view of the cameras was 87° × 58°, and the distance to the plants was such that the entire large mature plant would enter the field of view. With the considered setup and plants, the distance between the cameras and the plant rosette geometric center was about 25 cm. Lighting was provided by three 10W LED lights placed above the side cameras, with the light directed through white paper sheets for diffusion. To maintain consistent lighting conditions, the imaging chamber was enclosed with light-isolating curtains. To facilitate background filtering, the curtains and the chamber floor were designed with distinguishable colors. The point clouds captured by the four cameras were combined into a single point cloud using coordinate transformation, which was made possible by calibrating the camera positions before each imaging session.

RGBD images of the plants were captured using software provided by the camera manufacturer (Viewer, Intel RealSense, Santa Clara, California, USA) in .bag format. RGB color point cloud frames were extracted from the .bag files using the rs-convert tool (Intel RealSense SDK).

For accurate merging of point clouds achieved from different cameras, a calibration was performed before each recording session. During the calibration, an object with easily detectable features (Fig. 1c) was recorded by all the cameras. To maximize the visibility of the object and to fit between the point on different sides of the object seen by different cameras, the object was chosen to be a flat sheet—a paper sheet with format A6, with drawn marks to ease their detection by cameras and manual processing. To improve the accuracy by collecting more data, the calibration sheet was recorded 3–5 times in different locations and orientations. In each recorded point cloud, the points related to the calibration sheet were manually extracted from the rest of the points. Using all achieved point clouds, the space transformation parameters defining translation and rotation of the point clouds for each camera were calculated and stored. To calculate them, an optimization problem was solved. The cost function was the sum of differences between points in different point clouds. The optimization parameters were three translation and three rotation parameters in a space transformation matrix for each camera. The coordinate system of the top camera numbered 4 was taken as the reference, while the transformation matrices for the remaining cameras were calculated, resulting in 18 optimization parameters. Because of a large number of parameters and unstructured dataset including coordinated of partial point clouds resulting in multiple local minima, the genetic algorithm (GA) was used. A basic version of GA [26] was implemented with the number of generations 100, number of population 100 and mutation rate 0.1. The accurate definition of the cost function and implementation of the calibration algorithm are described in the Supplementary Information.

The accuracy of the calibration was estimated by labelling a reference point in the point clouds and measuring the distance between representations of this point in different point clouds. The reference point was chosen in an easily distinguishable location seen from all cameras for all positions of the calibrations sheet—the connection point between the sheet and its holder. Since the calibration sheet was located in different location and orientations in each calibration session, a number of the reference points in the measured volume inside the frame were available for accuracy estimation.

At the end of the experiment (23 days in NFT for experiment 1, and 21 days in NFT for experiments 2 and 3) after point cloud recording, the aerial parts of the plants were harvested, and their fresh weight (FW) was measured using a scale (DeltaRange PR5002, Mettler Toledo, Greifensee, Switzerland) on March 9, 2023, April 4, 2023 and May 8, 2023.

**Fig. 2:**
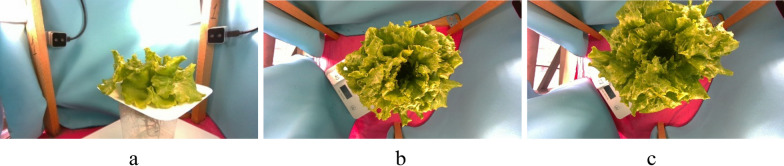
Examples of lettuce plants recorded in the experiment (**a**). The plant No. 40 (taken on 9 March 2023) with reference FW = 237.84g (**b**) and plant No. 72 (taken on 9 March 2023) with reference FW = 235.88g (**c**) have noticeably different sizes and leaf density because of high variability in the plant structure

The RGBD and point cloud frames recorded during this study were organized into a dataset named Pii (https://zenodo.org/deposit/8410252), which includes 402 3D point clouds with reference FW values ranging from 62.3 to 276.5 g (Fig. 2). Additional 3D images of these plants recorded throughout their growth were made as an attempt to measure plant biomass during growth. However, due to various uncertainties, the biomass measurements were not sufficiently accurate, hence, the images are available in the dataset, though they do not have FW reference values and were not considered in this study.

### Plant surface reconstruction

**Fig. 3:**
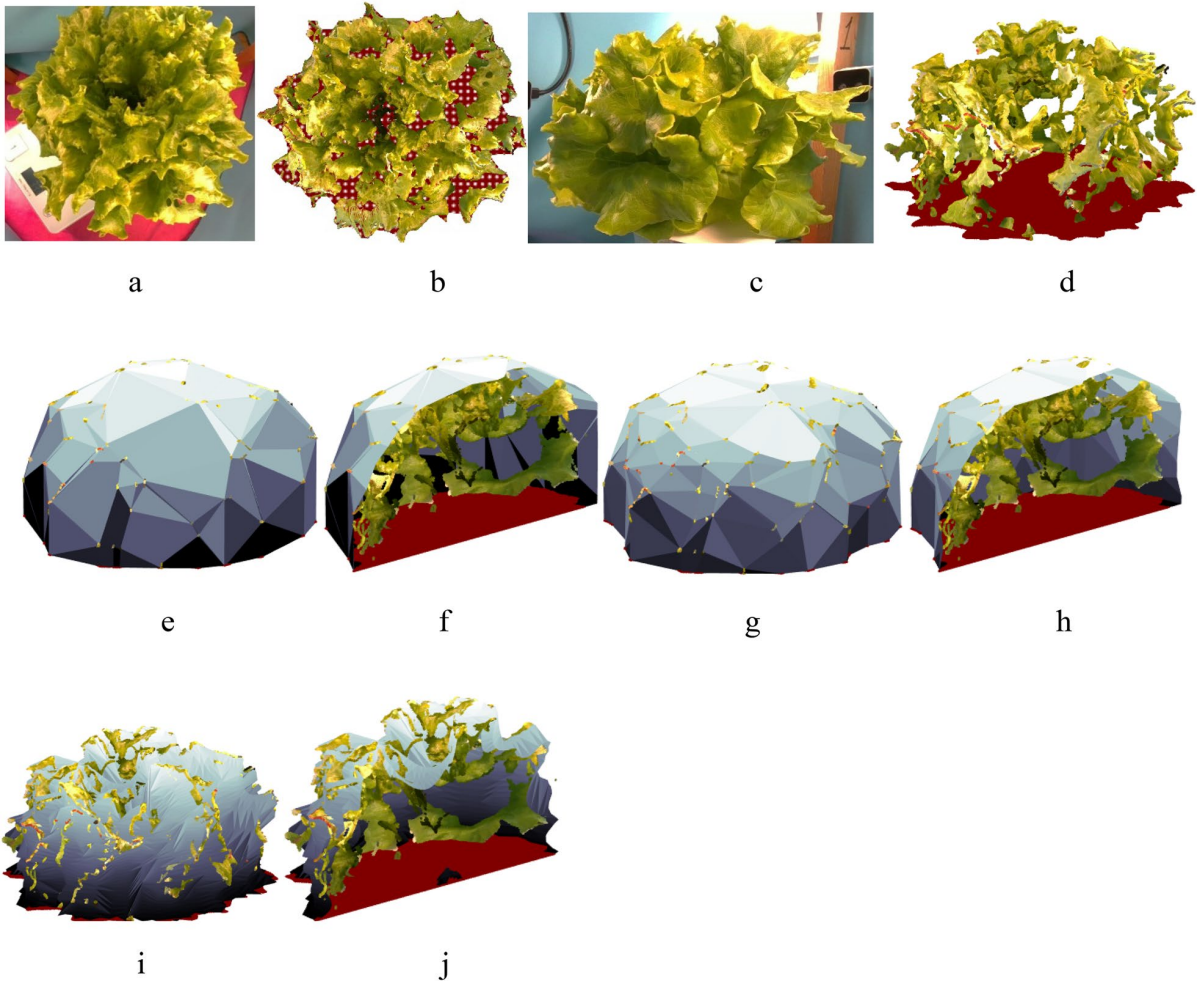
Example of a plant top (**a**) and side (**c**) views and their 3D point clouds recorded with the 3D cameras at top (**b**) and side (**d**) views. The point cloud surfaces reconstructed by Alpha shapes algorithm with the alpha parameters 0.2 (**e**) with a sectional view (**f**) and Ball Pivoting algorithm with the ball radius 5 cm (**g**) with a sectional view (**h**), and with the Vacuum package algorithm (**i**) with a sectional view (**j**)

Lettuce leaves have a complex structure, making 3D modeling with LIDAR or stereo 3D cameras challenging. In their mature stage, lettuce leaves are typically fractal-shaped, creating multiple hidden areas that are only visible to one RGB camera of a stereo setup. Additionally, the sharp angles of surfaces relative to LIDAR cameras prevent proper reflection of the projected light. The intricate interconnections between lettuce leaves also create narrow, deep spaces that are difficult for 3D cameras to capture. These factors lead to a lack of data and the formation of holes in the reconstructed surfaces (Fig. 3b, d). Moreover, the visual volume of the plant is strongly influenced by the rosette structure, meaning that plant density can vary widely, as shown in Fig. 2b and c. These challenges make accurate surface reconstruction particularly difficult. As a result, in this study, plant volume was defined as the volume enclosed by a tight hull around the plant model.

Two commonly used methods for surface reconstruction were tested: the Ball Pivoting algorithm [9, 27] and Alpha Shapes [28]. However, these algorithms failed to meet two conflicting requirements: accurately approximating the fractal-shaped lettuce surface with high detail while correctly closing the holes caused by missing data points. The algorithms either used small structural elements, which left holes in the surface (and sometimes created false structures inside the surface by connecting the holes), or they used larger elements to close the holes, resulting in rough surfaces with insufficient detail. To address this, the parameters of the tested algorithms were adjusted in this study to ensure that the surfaces were reconstructed without holes while maintaining the maximum level of detail. The following parameters were used: a 5 cm ball radius for the Ball Pivoting algorithm and an alpha parameter of 0.2 for Alpha Shapes (Fig. 3).

To address the issue of missing data, a specialized algorithm was developed for reconstructing the lettuce rosette surface, taking into account the unique geometry of the lettuce rosette. The algorithm was based on the assumption that most holes in the point cloud occur in the depressions between leaves or in areas hidden beneath the leaf surfaces. The proposed method uses a physical analogy: shrinking an elastic vacuum package around a solid body under external pressure during vacuum sealing.

**Fig. 4:**
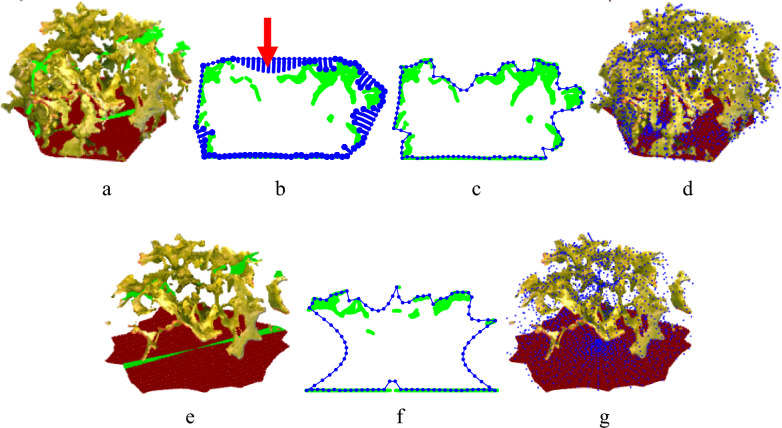
The proposed vacuum package method for surface reconstruction for a whole plant model (**a**, **b**, **c**, **d**) built from four views and a partial model (**e**, **f**, **g**) built from a top view: partition of a 3D point cloud to sectors (**a**, **e**), reconstructing the 2D surface curve (**b**), final tight hull of a sector (**c**, **f**) and the final point mesh of the reconstructed surface (**d**, **g**)

This vacuuming process for a 2D slice of the lettuce point cloud is illustrated in Fig. 4b. The elastic package represents the reconstructed body surface and is modeled by a set of package points (blue dots in Fig. 4b). Initially, the package points are positioned on the convex hull of the object. As the vacuuming process begins, the package shrinks until its points make contact with the body surface points (bold blue dots in Fig. 4b touching the green point cloud). However, in areas with missing data (large gaps, greater than 0.5 cm in size, as shown in Fig. 4b), the package continues to move inward through the holes under external pressure. The direction of this inward movement is perpendicular to the boundary points of the holes (indicated by the red arrow for a specific interval). The shrinking stops when the pressure balances with the elasticity of the package, which is represented by a condition where the maximum distance between the package points reaches a predefined threshold (bold blue dots in Fig. 4b, further from the green point cloud).

This algorithm creates a detailed surface similar to the Ball Pivoting algorithm, with the density of the package points matching the pivoting ball radius, while filling in all surface holes, regardless of their size (Fig. 4c). It also reconstructs the depressions between the leaves.

However, the full 3D plant model, which is built from three side views and one top view, is available only for part of the dataset. The remaining part of the dataset consists solely of top-view RGBD images. In cases with partial models, the information about the plant’s side structure may be obscured by the leaves, making it unavailable (Fig. 4e). In such cases, the vacuum package method is used to augment the plant structure, as shown in Fig. 4f.

The code for the vacuum package method, along with the data processing scripts used in this study, are available in the Supplementary Information.

To keep the surface reconstruction algorithm simple, only the two-dimensional version of the vacuum package method was implemented in this study, while the development of a full 3D algorithm was beyond the scope of this work. To apply the algorithm, the plant point cloud was divided into 24 equal angle sectors by planes passing through a central axis as shown in Fig. 4a and c. The central axis was the Z axis in the coordinate systems defined as show in Fig. 6a and b: the XY was the plant base surface, X axis was defined as the X axis of the camera 4, the origin was located on the XY plane in the geometric center of the point cloud. To turn the points inside the sectors into a planar object, the points were projected on one of the plains defining the sector. A tight hull with 200 points was generated for each sector.

The final surface points for each sector were then triangulated to create a surface mesh. The plant volume defined by the hull was calculated by summing the volumes of the individual sectors. The sector volume was approximated using trapezoidal rule for integration

1 $${V=\sum_{k=1}^{24}{V}_{k}, V}_{k}=\sum_{i=1}^{200}\frac{{y}_{i+1}+{y}_{i}}{2}d{S}_{i}, { dS}_{i}=\pi \left({x}_{i+1}^{2}-{x}_{i}^{2}\right)$$

where *V* is the total plant volume estimation, *V*_*k*_ is a volume estimation for the sector *k*, *y*_*i*+*1*_, *y*_*i*_, *x*_*i*+*1*_ and *x*_*i*_ are the y and x coordinates of the points *i* + *1* and *i* of the sector hull, *dS*_*i*_ is the area of the *i* part of the sector *k*.

In this study, two parameters of the method, the number of the sectors, and the number of the hull points were found empirically for this specific dataset. The tight hull with 200 points for each sector resulted in a maximum distance of about 5 mm between the hull points. The number of sectors was taken such that each sector was assumed to be thin enough to be treated as a planar object (Fig. 4b). For other datasets, they must be fitted according to the point and hole density in the point clouds.

### Plant structure characterization

The shape of the plant rosette can vary significantly between individual plants (Fig. 2b and c), which strongly impacts the ability to estimate the plant’s volume. A hull covering a plant with loosely arranged leaves (similar to a rose) will enclose more volume than a plant with the same mass but tighter leaves (like a cabbage). However, the close hull generated by the Vacuum algorithm allows for the characterization of leaf density near the rosette surface.

**Fig. 5:**
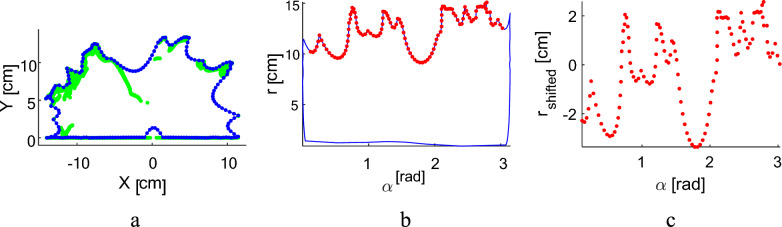
Transformation of the reconstructed plant section hull (**a**, blue dots) to a continuous plant surface profile. The hull is transformed to the polar coordinates, only the plant upper part is taken (**b**, red dots) and the points are shifted by their average value (**c**)

To quantify the leaf distribution, the rosette profiles for all sections were used (an example is shown in Fig. 5a). To account for the deepening between leaves, the Cartesian coordinates (Fig. 5a) were converted into polar coordinates (r, α), where r represents the distance of the package points from the center of the geometric lettuce point cloud (point (0,0) in Fig. 5a), and α represents the azimuth angle (Fig. 5b). Only points within the range 0.1 < α < π – 0.1 and r > 0.2 cm were considered.

To eliminate the influence of leaf height, the profile was shifted by its average value. The resulting profiles for all sections were then concatenated into a continuous surface profile that represents the entire plant.

Several geometric features were calculated for the plant surface profile. The profile exhibits an oscillating wave pattern, where the wave crests correspond to the tips of the leaves, and the troughs correspond to the depressions between the leaves. The average wave amplitude (Amp), calculated as the difference between the crests and troughs, was used to characterize the average leaf length. Additionally, the plant height (Height) was calculated as the 95 percentile of the point cloud height for the entire plant.

### Fresh weight prediction by regression

The plant point cloud only represents the outer surface of the plant without capturing the complex inner rosette structure. Additionally, as mentioned earlier, the 3D models of the plant contain multiple holes. These two factors prevent direct calculation of the plant volume using the currently available sensors. Consequently, the plant FW cannot be accurately calculated by assuming a constant leaf density.

The volume enclosed by the reconstructed surface can serve as an estimator for FW. While the relation between the FW and the leave length, density and plant height can be assumed, the influence of their estimators Amp and Height can be used to refine the estimated volume by accounting for the leaf structure. However, this influence is not clear and was tested in this study in simple and multiple linear regression models predicting FW.

### Model accuracy

Root mean square error (RMSE) and normalized RMSE (NRMSE) were used to estimate the accuracy of the models, where ${y}_{i}$ was the reference FW and $\widehat{{y}_{i}}$ was the predicted FW value.

2 $$RMSE=\sqrt{\frac{{\sum }_{i=1}^{N}{\left({y}_{i}-\widehat{{y}_{i}}\right)}^{2}}{N}}$$

3 $$NRMSE=\sqrt{\frac{{\sum }_{i=1}^{N}{\left({y}_{i}-\widehat{{y}_{i}}\right)}^{2}}{{\sum }_{i=1}^{N}{\left({y}_{i}\right)}^{2}}}$$

To perform validation for the regression models, a tenfold validation was applied with averaging the RMSE and NRMSE.

## Results

**Fig. 6:**
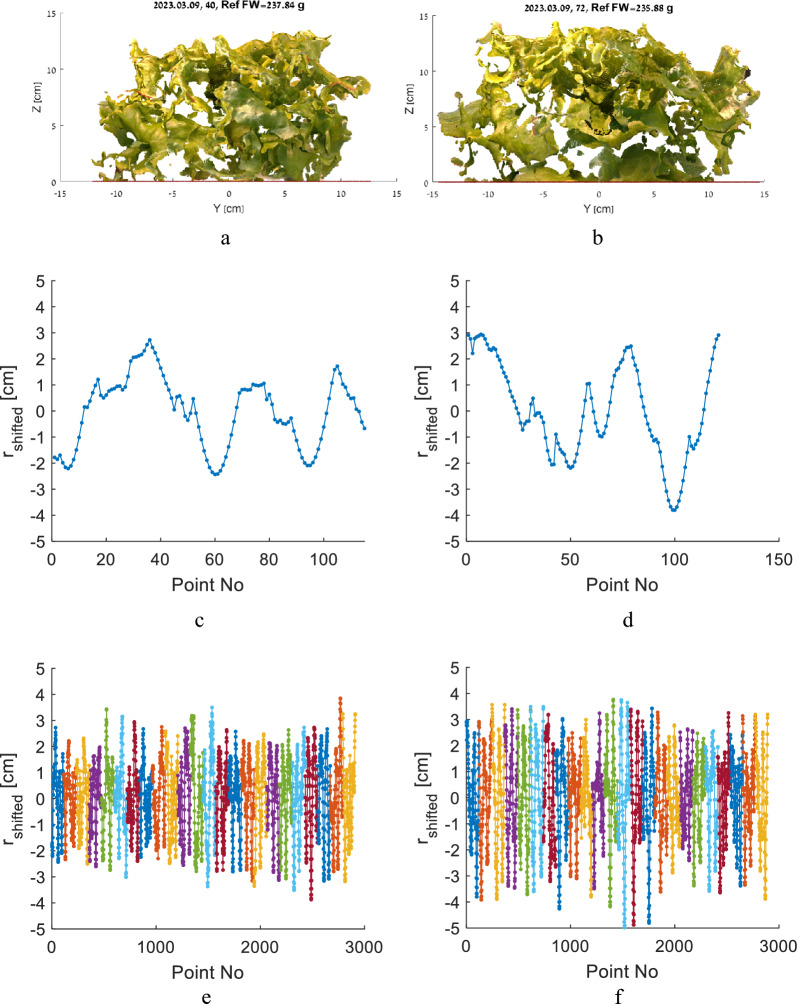
Comparison of the views and features of two plants No. 40 (**a**, **c**, **e**) and No. 72 (**b**, **d**, **f**) from Fig. 2b and c with similar FW and significantly different estimated volume: the side view of the recorded point clouds (**a**, **b**), an example of the plant surface profile of one section (**c**, **d**), the plant surface profile for all plant sections represented by different colors (**e**, **f**)

The features of the plants No. 40 and No. 72 (Fig. 2b and c) are presented and compared in Fig. 6a and b.

The reference FW of these plants are close, 237.84 g and 235.88 g for Plant No. 40 and 72, hence, we assumed that actual plant canopy volumes are close because of uniform density of the lettuce leaves. However, the difference in rosette dimensions is clearly evident from the plant point clouds, and the estimated V and Amp were significantly different: V was 3.155 l and 4.33 l, and Amp was 2.4 cm and 2.9 cm for Plant No. 40 and 72 respectively. The Height was close: 2.4 cm and 2.9 cm.

**Fig. 7:**
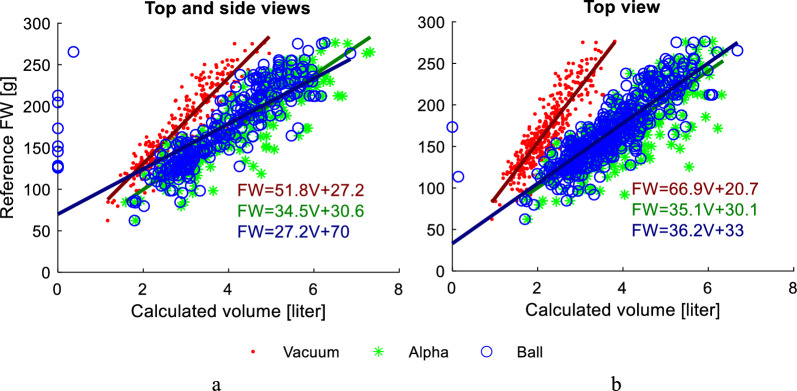
Correlation between the reference plant FW and estimated plant volume for the Ball pivoting, Alpha shapes and proposed Vacuum packaging surface reconstruction methods for the 3D models achieved from the top view images (**a**) and the top and three side view images (**b**)

The correlation between the calculated plant volume and the reference plant FW, based on simple regression, is shown in Fig. 7 and Table 1. The correlation is analyzed with respect to the number of 3D images used and different surface reconstruction methods.

**Table 1:** Accuracy of FW prediction (RMSE, NRMSE, R^2^) and simple linear regression models (FW(V), where V is the calculated volume in liters) for different surface reconstruction methods

	Vacuum	Alpha shape	Ball pivoting
*Top and three side view images*			
RMSE, g	**18.2**	21.7	30.2
NRMSE, %	**10.1**	12.0	16.7
FW(V)	51.8V + 27.2	34.5V + 30.6	27.2V + 70
R^2^	0.85	0.78	0.58
*Top view 3D image*			
RMSE, g	**18.9**	23.5	21.4
NRMSE, %	**10.9**	13.5	12.3
FW(V)	66.9V + 20.7	35.1V + 30.1	36.2V + 33
R^2^	0.79	0.68	0.73

The minimum errors are highlighted in bold

The accuracy of FW estimation was similar across different surface reconstruction algorithms, with root mean square errors (RMSE) ranging from 18.9 to 23.5 g. However, the ratio between the predicted FW and the calculated volume (i.e., the slope of the regression line) differed significantly between surface reconstruction methods, indicating variations in the level of detail in the surface reconstructions.

FW prediction with three side 3D images in addition to the top 3D image had higher accuracy for the Vacuum package and Alpha shape algorithms.

**Fig. 8:**
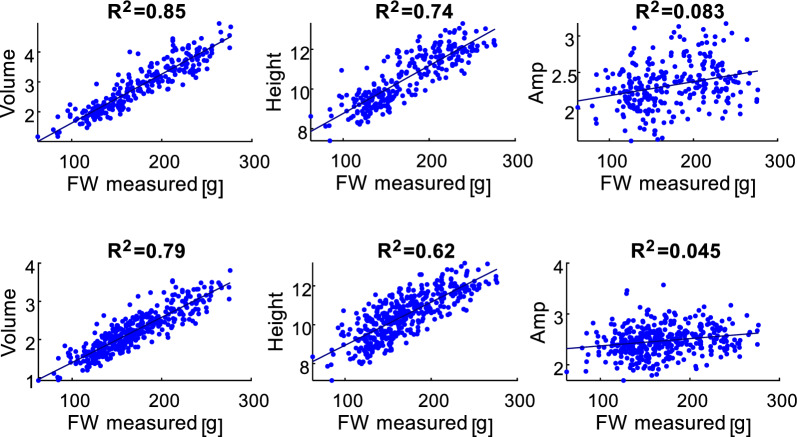
Correlations between the measured FW and geometric features for the top and three side view 3D images (upper row) and top view 3D image (lower row)

The correlation between the measured FW and the plant features extracted from the plant surface profile is shown in Fig. 8 for simple linear regression and in Table 2 for multiple linear regression. In the simple regression, V exhibits the strongest correlation with FW. In the multiple regression models, all equations that achieve the highest accuracy include V.

**Table 2:** FW prediction multiple linear regression models with different parameter number (PN) for top only and top and three side view 3D images

PN	R^2^	RMSE, g	NRMSE, %	FW
*Top and three side view 3D images*				
1	0.85	18.2	10.1%	27.2 + 51.8·V
	0.74	23.8	13.2%	− 149.6 + 30.7·Height
	0.08	44.6	24.7%	74.5 + 43.0·Amp
2	0.86	17.6	9.7%	57.8 + 54.3·V-16.2·Amp
	0.85	17.9	9.9%	− 6.9 + 44.3·V + 5.2·Height
	0.74	23.7	13.1%	− 142.1 + 31.2·Height-5.4·Amp
3	0.86	17.3	9.6%	23.7 + 46.8·V + 5.2·Height-16.2·Amp
*Top view 3D images*				
1	0.79	18.8	10.9%	20.7 + 66.9·V
	0.62	25.3	14.6%	− 125.2 + 28.1·Height
	0.04	40.4	23.2%	91.1 + 31.5·Amp
2	0.8	18.2	10.5%	58.3 + 70.2·V-18.2·Amp
	0.8	18.5	10.7%	− 14.5 + 57.4·V + 5.4·Height
	0.63	25.0	14.4%	− 157.0 + 27.6·Height + 15.1·Amp
3	0.81	18.1	10.4%	30.5 + 63.7·V + 3.4·Height-15.6·Amp

Although the inclusion of additional features in the prediction equations can improve prediction accuracy, a clear influence of these features was not observed for the dataset used in this study.

**Fig. 9:**
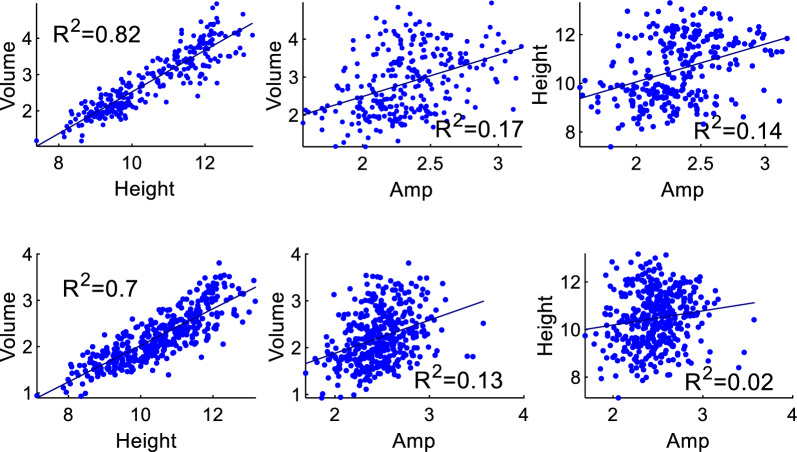
Correlation between the geometric features extracted from the plant surface profile for top and three side view 3D images (upper row) and top view 3D images (lower row)

The correlation between the geometric features is presented in Fig. 9. The V and Height show a strong correlation, while the Amp is not correlated with V.

The calibration accuracy was 2.6 mm on the March 9, 2023 recording session and 6.2 mm on the April 4, 2023 session.

## Discussion

Using the proposed vacuum package method with only top-view 3D images, FW was estimated with an RMSE of 18.9 g and an NRMSE of 10.8% for the dataset collected in this study, where FW ranged from 62.3 to 276.5 g. These errors are comparable to or lower than RMSE values of 25.3 g [19], 27.85 g [17], or an NRMSE of 15.63% [18], and RRMSE values of 0.193 [9], all achieved for the Autonomous Greenhouse Challenge [15] dataset, which also consisted of only top-view 3D images and had an FW range between 1.4 and 459.7 g. When applied to the Autonomous Greenhouse Challenge dataset, the proposed method achieved an RMSE of 49.6 g and an NRMSE of 24.1% for a simple regression model and 46.2 g and 22.4% for a multiple regression model with all three considered parameters. This high error could be explained by different qualities of the datasets which results from different types of the 3D cameras used for their creating: the short range stereo visual camera (D405, Intel RealSense, California, USA) used in this study created point clouds with missing data in the depressions between the leaves, while the middle range stereo IR camera (D415, Intel RealSense, California, USA) used for the Autonomous Greenhouse Challenge dataset created continuous point clouds with smoothened depressions between the leaves.

The surface reconstruction using the proposed vacuum packaging method was specifically developed for surfaces with multiple curved interconnected folds and deep depressions, such as those seen in lettuce rosettes. According to Table 1, this method improves FW prediction compared to other tested methods, even when the lower part of the rosette is hidden. Regression models based on the vacuum method (Table 1) had a higher slope coefficient, meaning that V was less overestimated by the vacuum method than by the other methods. The y-intercept coefficient was closer to zero in the vacuum method models, reflecting a more accurate relationship between V and FW.

Geometric features extracted from the plant surface profile can be used to characterize leaf density and structure. These features provide insights into the average number of leaves in rosette cross-sections and the size of the depressions between leaf tips. While these features are implicitly incorporated in CNNs used for lettuce monitoring, their value is difficult to extract and lacks a physical explanation for direct application.

According to Table 2, the most significant feature in predicting FW is V. The Amp feature, which characterizes the size of the depressions between leaves, improves FW prediction by accounting for voids within the total volume estimation. This physical interpretation corresponds to the negative correlation of Amp in FW prediction models that include V. The Height feature is strongly correlated with V, meaning its inclusion in the FW prediction model does not significantly improve accuracy. However, Height can be useful for characterizing plant structure, such as distinguishing between tall and short plants with similar estimated volumes. Combinations of the features in the regression models were tested, however, no significant improvements were received, while the complexity of the equations increased and explainability of the models worthened.

The errors of the 3D cameras and the calibration look small relatively to a typical size of the lettuce canopy. Its influence on the FW prediction accuracy should be analyzed in the further study. The calibration sheet used in this study was seen by the camera in majority of cases, but to improve the calibration process, a devise with easily and strictly distinguishable objects seen by all the cameras should be constructed. According to the experience from this study, the distinguishable objects should be flat to avoid uncertainty when it is modeled by cameras seeing different sides of the object, and as small as possible to decrease the uncertainty when only a part of the object is modelled.

The volume calculation method has a clear physical basis. However, because the internal structure of the lettuce rosette composed of leaves and the voids between them cannot be captured with the current 3D camera technology, the true volume must be estimated using models. In this study, linear regression models were applied. Since only one dataset with a limited range of plant weights and growth stages was used, the regression models may not be generalizable to other datasets collected in different environments or for different lettuce varieties. More diverse datasets with accurate point clouds are required to develop more universal methods for FW estimation during plant growth. Additionally, the parameters of the method, such as the resolution of surface points and the maximum distance between points, should be adjusted according to the specific geometric characteristics of the plant and the resolution of the 3D camera.

## Conclusions

In this study, a new method for reconstructing lettuce surfaces for fresh weight estimation was proposed. This method, with a direct physical basis, offers an alternative to neural network-based approaches, which still suffer from limited datasets. New features characterizing lettuce leaf density and structure were proposed for breeding and fresh weight estimation. A setup for 3D recording of lettuce and a corresponding dataset of lettuce 3D point clouds were introduced. For future research, additional and more varied data need to be collected to further refine and validate the proposed methods.

### Supplementary information

The code used at this study is available at https://github.com/VicB18/LettuceFW (accessed on 1 November 2024).

## Data Availability

Data used in this study and developed models are available on Zenodo storage service https://zenodo.org/records/8410252.

## Abbreviations

FW: Fresh weight
(N)RMSE: (Normalized) root mean square error
RGBD: Red, green, blue, depth
